# Accelerating antibody discovery and optimization with high-throughput experimentation and machine learning

**DOI:** 10.1186/s12929-025-01141-x

**Published:** 2025-05-09

**Authors:** Ryo Matsunaga, Kouhei Tsumoto

**Affiliations:** 1 Department of Bioengineering, School of Engineering, The University of Tokyo, Tokyo, 113-8656 Japan; 2 Department of Chemistry and Biotechnology, School of Engineering, The University of Tokyo, Tokyo, 113-8656 Japan; 3 The Institute of Medical Science, The University of Tokyo, Tokyo, 108-8639 Japan

**Keywords:** Antibody therapeutics, Machine learning, Data-driven design, Antibody design, Computational antibody engineering

## Abstract

The integration of high-throughput experimentation and machine learning is transforming data-driven antibody engineering, revolutionizing the discovery and optimization of antibody therapeutics. These approaches employ extensive datasets comprising antibody sequences, structures, and functional properties to train predictive models that enable rational design. This review highlights the significant advancements in data acquisition and feature extraction, emphasizing the necessity of capturing both sequence and structural information. We illustrate how machine learning models, including protein language models, are used not only to enhance affinity but also to optimize other crucial therapeutic properties, such as specificity, stability, viscosity, and manufacturability. Furthermore, we provide practical examples and case studies to demonstrate how the synergy between experimental and computational approaches accelerates antibody engineering. Finally, this review discusses the remaining challenges in fully realizing the potential of artificial intelligence (AI)-powered antibody discovery pipelines to expedite therapeutic development.

## Background

Antibody therapeutics have become increasingly important over the past few decades, highlighting the crucial role of antibodies in immune responses. These biomolecules have become a major focus in drug development owing to their unique specificity and versatility. Initially used primarily to treat cancers and autoimmune disorders, the use of antibodies as drugs has rapidly expanded in recent years. They are currently being actively investigated for the treatment of several diseases, including infectious diseases [1] and allergies [2], which is driving substantial growth of this therapeutic modality.

The global therapeutic antibody market is experiencing rapid growth driven by an aging population, an increase in chronic diseases, and a shift toward biologics that provide targeted therapeutic mechanisms. Emerging economies are witnessing a surge in demand for antibody drugs, driven by rising healthcare expenditures and improved access to advanced treatments. Consequently, the antibody therapeutic pipeline continues to flourish, with more than 100 new candidates currently undergoing late-stage clinical development [3].

A significant challenge faced by the pharmaceutical industry is the accelerated development of novel, highly effective, and safe antibody drugs to meet the increasing global demand for such treatments [4]. Rapid optimization of lead candidates with improved therapeutic profiles is crucial for delivering innovative treatments to patients worldwide [5–7]. Conventional antibody development in the laboratory has been hampered by significant constraints in terms of throughput, cost, and exploration of vast candidate spaces. Traditional approaches such as hybridoma technology require laborious procedures, including the isolation of antibody-producing cells, cloning, and screening, and often require several months or years to identify lead candidates. A key challenge lies in the need to evaluate multiple antibody candidates to identify those with the desired antigen specificity. However, these experimental techniques are limited by their ability to investigate diverse antibody sequences and structures. Characterization of antibody candidates requires various low-throughput experimental assays, such as binding affinity measurements and X-ray crystallography studies, to assess critical properties, such as antigen recognition, affinity, and specificity. This bottleneck hinders the rapid evaluation of large antibody libraries.

Antibody affinity maturation, which increases antibody binding strength, is crucial for obtaining optimal therapeutic candidates. Conventional methods have significant limitations in efficiently exploring sequence spaces for improved variants. The evaluation of physicochemical properties and formulation stability, which are essential for manufacturing and therapeutic applications, is equally important. This is also a labor-intensive process that relies on empirical experimentation.

**Fig. 1:**
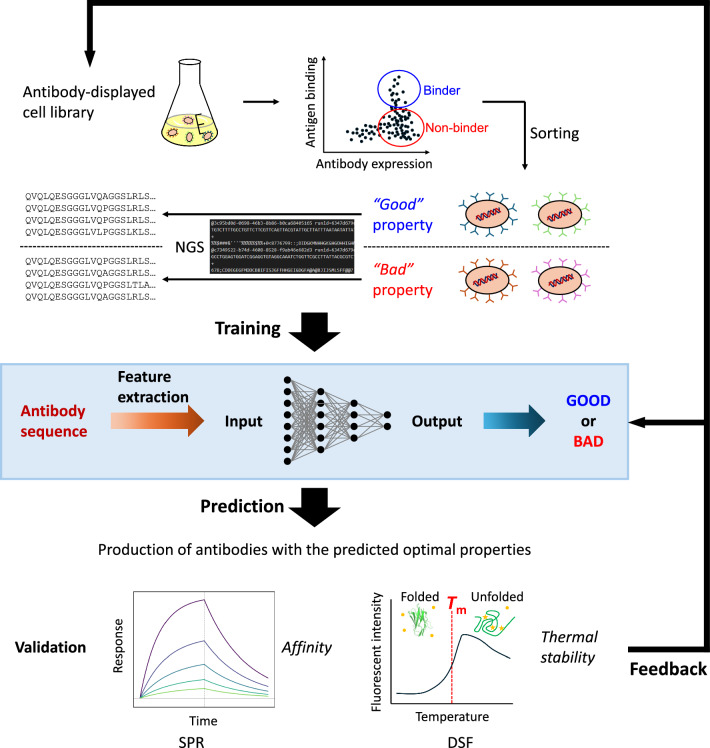
Concept of data-driven antibody design. An overview of a typical process is presented. This process encompasses several critical stages, beginning with the construction of diverse antibody libraries via methods such as yeast or phage display techniques. This is followed by high-throughput screening, which uses techniques such as FACS or biopanning to identify cells that produce antibodies with the desired properties. NGS is then employed to reveal DNA sequences of antibody-encoding genes from these selected cells. The resulting sequence data are transformed into numerical features via methods such as protein language models or graph neural networks. These features, in conjunction with experimental data, are employed to train an ML model with the objective of establishing relationships between antibody sequences and their properties. The trained model then predicts the properties of new antibody sequences and identifies promising candidates for further development. These predicted optimal antibodies are then produced, and their properties are validated through experimental assays. In some cases, the data obtained from these experimental assays are fed back into the library design or the ML model to refine its predictive capabilities, creating a closed-loop optimization process

Recent advances in high-throughput experiments and machine learning (ML) have led to the emergence of data-driven approaches as powerful paradigms for accelerating antibody development [8–12]. These methods utilize large-scale datasets, encompassing antibody sequences, structures, and binding assay readouts, in conjunction with ML algorithms to facilitate the rational design and optimization of therapeutic antibody candidates (Fig. 1). In contrast to traditional empirical and trial-and-error approaches, data-driven engineering of antibodies offers a more systematic and efficient framework for antibody discovery and the optimization of lead candidate antibodies. Crucially, this approach goes beyond merely improving affinity; by capturing intricate sequence–structure–function relationships, data-driven methods can predict and optimize various properties relevant to developability, including affinity, cross-reactivity, and physicochemical stability, without exhaustive empirical screening.

This review highlights the recent advancements in data-driven antibody engineering, focusing on the crucial role of high-throughput experimental data acquisition in these developments. It explores the key components of the field, including data acquisition techniques, computational analysis of antibody sequences and structures, and ML models for predicting both affinity and comprehensive developability profiles. The integration of large-scale data with advanced ML models offers an efficient framework for accelerating antibody development, which has been extensively reviewed [8, 10, 13–17]. Unlike previous reviews, we emphasized the synergy between high-throughput experimentation and computational modeling, particularly for experimental scientists seeking to leverage this approach. This synergy supports rational in silico optimization and significantly improves empirical methods. Finally, we address the challenges ahead and potential of AI in antibody discovery for on-demand therapeutic antibody design and development.

## High-throughput data acquisition methodology

First, we reviewed the experimental techniques required for data-driven antibody design. To provide a context for the specific antibody design applications discussed in a later section, we briefly describe this technology and its features.

### Next-generation sequencing (NGS) technologies

Next-generation sequencing (NGS) technologies have revolutionized antibody repertoire analysis by enabling massive parallel high-throughput sequencing, providing a detailed view of diverse antibody repertoires [18]. Different NGS platforms, such as Illumina [19], Ion Torrent [20], Pacific Biosciences (PacBio) [21], and Oxford Nanopore [22], offer unique advantages in terms of read length, accuracy, and throughput. These technologies have facilitated the identification of rare clones within antibody repertoires and enabled the study of antibody lineage evolution during affinity maturation [23–26]. Long-read sequencing is particularly important for capturing complete variable regions and characterizing complementarity determining regions (CDRs) with high precision [23]. Furthermore, optimized library preparation protocols, incorporating antibody-specific amplification, target enrichment, and unique molecular identifiers, have significantly enhanced the efficiency of NGS for antibody analysis [24, 26]. Coupled with the development of tailored bioinformatics methods [25], these advancements provide unprecedented depth of analysis, opening new avenues for understanding the complexities of antibody repertoires [27]. Specifically, BCR sequencing, a specialized application of NGS, allows for the detailed analysis of B-cell receptor diversity, including the identification of paired heavy and light chain sequences from individual B cells [23–25]. This information is crucial for understanding the antibody repertoire and identifying antibodies with specific binding properties, which can be further developed into therapeutics.

### Display technologies for antibody library screening

Antibody display technologies, in conjunction with techniques such as biopanning and fluorescence-activated cell sorting (FACS), have become invaluable for the high-throughput screening of antibody libraries (Table 1). These technologies help identify the sequences of rare antibody binders from vast sequence spaces. Phage display technology facilitates the expression of antibody fragments on phage coat proteins, which can be enriched against immobilized antigens [28–30]. This allowed the screening of libraries larger than 10^10^ in size. Yeast displays employ yeast cells to express antibodies on their surfaces, which can then be sorted via FACS to detect fluorescent antigens [31–34]. This approach takes advantage of eukaryotic protein folding and enables the exploration of libraries up to 10^9^ in size. Mammalian cell displays detect the expression of antibodies on mammalian cell surfaces [35, 36]. This approach offers a screening environment that closely mimics natural antibody conditions and post-translational modifications.

**Table 1:** Representative methodologies for experimental affinity screening systems

Methodology	Principle	Advantages	Limitations	Typical Library Size
Phage Display	Antibody fragments are displayed on phage coat proteins, enabling selection against immobilized antigens	High throughput, large library sizes, amenable to in vitro evolution	May require specialized equipment, potential to obtain false positives due to phage surface interactions	< 10^11^
Yeast Display	Antibodies are displayed on the surface of yeast cells, allowing FACS-based sorting for antigen binding	Eukaryotic protein folding, high throughput screening, amenable to genetic manipulation	Limited by the size of the yeast cell surface, requires specific yeast strains	< 10^9^
Mammalian Cell Display	Antibodies are expressed on the surface of mammalian cells, providing a native-like environment for screening	Accurate representation of antibody function, allows for post-translational modifications	Lower throughput than phage or yeast display, requires specialized cell lines	< 10^8^
Ribosome Display	Antibody-mRNA complexes are formed and stabilized on ribosomes, allowing for selection based on antibody-antigen binding	Cell-free system, high throughput, large library sizes, suitable for toxic or difficult-to-express proteins, amenable to in vitro evolution	mRNA-ribosome-protein complexes can be unstable and prone to dissociation during selection. mRNA may be degraded by nucleases in the reaction mixture	< 10^15^

In addition, cell-free systems such as ribosome or cDNA displays facilitate the rapid exploration of sequence diversity without the need for transformation or transfection [37, 38]. These technologies enable the enrichment of antibodies that bind to antigens with desirable characteristics such as high specificity and affinity. Furthermore, the advent of techniques such as microfluidic screening and droplet-based microfluidics has revolutionized the field by enabling high-throughput screening of antibody libraries at a single-clone resolution [39]. A combination of different display platforms and advanced screening methods allows access to a wide array of antibody sequences, thereby paving the way for the identification of optimal antibodies for therapeutic applications.

### High-throughput analysis of antigen‒antibody interactions

The comprehensive characterization of antigen-binding properties is essential after the initial screening of antibody libraries to identify lead candidates [40]. High-throughput techniques, such as enzyme-linked immunosorbent assay (ELISA), bio-layer interferometry (BLI) and surface plasmon resonance (SPR), offer quantitative assessment of antibody‒antigen interactions at the single-clone level, providing valuable insights into kinetics, affinity, and specificity (Table 2).

**Table 2:** Representative methodologies for experimental affinity validation systems

Methodology	Principle	Advantages	Limitations	Throughput	Kinetic Data?
Enzyme-Linked Immunosorbent Assay (ELISA)	Antigen is immobilized on a solid phase, and antibody binding is detected using an enzyme-linked secondary antibody	Versatile, well-established, relatively low cost	Not as good compared to other methods for quantitation, potential for high background signal	Moderate to High (96-well format common)	No
Single-Molecule Counting (SMC)	Fluorescently labeled antibodies are used to detect and quantify individual antibody-antigen complexes	High sensitivity (sub-pg/mL), quantitative, allows multiplexing, faster read times than ELISA	Requires specialized equipment, not usually used for antigen–antibody affinity analysis	High (384-well format)	No
Bio-layer Interferometry (BLI)	Measures changes in interference patterns caused by antibody-antigen binding on a sensor	Label-free real-time analysis, suitable for crude samples	Requires specialized equipment, lower throughput than ELISA	Low to Moderate (96 or 384-well format common)	Yes
Surface Plasmon Resonance (SPR)	Detects changes in refractive index at a sensor surface upon antibody-antigen binding	Label-free real-time analysis, highly sensitive	Requires specialized equipment and expertise, higher cost	Low (typically single-channel, but high-throughput systems exist)	Yes

Although ELISA is a widely used and cost-effective plate-based method for measuring antibody binding, it cannot provide kinetic information, unlike BLI and SPR. BLI is a label-free technique that measures interference patterns resulting from the interaction between antibodies on biosensors and antigens in solution [41]. This allowed for real-time analysis of up to 96 simultaneous interactions. Taking advantage of the ease of measurement, combined with a cell-free expression system, we developed FASTIA, a system that can analyze the binding characteristics of dozens of antibody variants in two days [42]. Similarly, SPR is another label-free method that detects changes in the refractive index at the sensor surface upon antigen‒antibody binding. This enabled screening of antibody clones in kinetic assays and epitope binning. Until recently, the throughput of SPR measurements was limited. However, in recent years, some models have become capable of simultaneously measuring multiple samples. Recent advancements have led to the development of high-throughput systems capable of simultaneously measuring hundreds of antibody‒antigen interactions. For example, systems such as BreviA [43] utilize instruments capable of measuring 384 interactions simultaneously. These high-throughput systems generate large datasets of binding kinetics and affinity, which are essential for training and validating machine learning models used in data-driven antibody design.

Instead of using a specific device dedicated to measuring interactions, an ingenious system was proposed that allows the ribosomal display of antibodies on an Illumina flow cell to measure 10^8^ interactions with antigens [37]; however the accuracy may be limited by incomplete control of the antigen supply and dissociation.

These biophysical methods yield detailed binding-affinity data that are crucial for the development of lead antibodies. These assays enable the efficient screening of extensive antibody collections when integrated with robotic systems and automated liquid handling. These high-throughput characterization techniques accelerate the identification of prime therapeutic candidates and support targeted antibody engineering on the basis of thorough antigen-binding analyses.

### High-throughput stability analysis

Evaluating the physicochemical stability of antibodies via high-throughput methods is essential for assessing their developability and manufacturing feasibility. Techniques such as differential scanning calorimetry (DSC) offer in-depth thermodynamic stability profiles [44], but are limited by their low throughput, which restricts their widespread use in antibody engineering. In contrast, differential scanning fluorimetry (DSF) allows rapid assessment of antibody stability by detecting changes in fluorescence as proteins unfold, indicating the exposure of hydrophobic regions. This method facilitates rapid ranking of antibody stability in a plate-based format [45]. By refining the methodology for high-throughput interaction analysis described previously [43], we developed a novel system that permits the simultaneous production of antibodies, sequencing via nanopore technology, and acquisition of thermal stability data for hundreds of antibodies via DSF [46]. Instead of directly measuring denaturation, activity-based stability assays enable the comparison of the relative stabilities of various antibody variants by assessing their retained activity after exposure to thermal or chemical stress [47].

The integration of these high-throughput methods enables antibody engineers to screen and prioritize several candidate antibodies efficiently on the basis of their physicochemical properties. This streamlines the selection of stable leads for further refinement and development of manufacturing processes.

## Feature extraction from antibodies for ML

To perform ML of antibodies via high-throughput experimental data, such as those described above, each antibody must be represented as numerical data. This section briefly outlines the methodology used to obtain the representation.

### Feature extraction from sequences

Sequence-based featurization plays a crucial role in translating primary antibody structures into informative input representations for use in ML models. The most basic approach is one-hot encoding [29, 34, 35], which constructs a binary vector indicating the presence or absence of each amino acid at each position in the sequence. However, simple one-hot encoding fails to capture any biochemical relationships between residues. More advanced featurization strategies have been developed to incorporate biophysical and structural properties. For example, encoding schemes based on the physicochemical properties of residues [34], such as hydrophobicity, charge, and size, can provide more comprehensive representations that accurately reflect sequence‒structure relationships. Additionally, statistical metrics, such as position-specific scoring matrices (PSSMs) derived from multiple sequence alignments, offer insights into evolutionarily conserved patterns [48, 49].

Recently, language models pre-trained on massive protein sequence databases have emerged as powerful featurizers (Table 3). These protein language models, which are analogous to text-based models such as Long Short Term Memory (LSTM) [50] and Bidirectional Encoder Representations from Transformers (BERT) [51], learn the contextual representations of amino acid sequences through self-supervised training. When applied to antibody sequences, they can capture complex patterns and long-range dependencies relevant to antibody behavior.

**Table 3:** Representative pre-trained protein language models that can be used for the extraction of antibody features

Model Name	Architecture	Training Data	Validated Applications	References
UniRep	Multiplicative Long Short-Term Memory (mLSTM)	UniRef50 sequences [52]	Predicting binding affinity, stability, and expression levels	[53]
ESM-1b	Transformer	UniRef50 sequences	Secondary structure prediction, contact map prediction, remote homology detection	[55]
ESM-2	Transformer	UniRef50 and UniRef90 sequences	Atomic-level protein structure prediction, protein function prediction	[56]
ESM-IF1	Transformer with Geometric Vector Perceptron (GVP) layers	Sequences and structures from CATH [81], UniRef50 sequences and their predicted structures using AlphaFold2	Inverse protein folding (predicting sequence from structure)	[73]
ESM-3	Bidirectional transformer	Sequences from UniRef, MGnify [82], JGI [82, 83], OAS [59], Sequences and structures from PDB, AlphaFoldDB, ESMAtlas	Multimodal protein generation (sequence, structure, function), protein design	[64]
AntiBERTy	BERT	Natural antibody sequences [84, 85]	Understanding antibody affinity maturation process, generating diverse antibody sequences	[57]
AbLang	Transformer	OAS	Completing antibody sequences, identifying functionally relevant mutations, designing novel antibodies	[58]
IgBert	BERT	OAS (unpaired + paired)	Antibody sequence recovery, binding affinity prediction	[60]
IgT5	Text-to-Text Transfer Transformer (T5)	OAS (unpaired + paired)	Antibody sequence recovery, binding affinity prediction	[60]

A common approach for utilizing PLMs is to compute embeddings for each residue in the antibody sequence. These residue-level embeddings, which are high-dimensional vectors representing the contextual information of each amino acid, can then be aggregated (e.g., by averaging) to obtain a fixed-length vector representation of the entire antibody sequence or specific regions such as CDRs. This vector can be used as input for downstream machine learning tasks, such as predicting binding affinity, specificity, or developability. However, it is important to note that this is not the only way to utilize PLM-derived features. Other approaches include using the residue-level embeddings directly as inputs for convolutional neural networks or graph neural networks, or employing attention mechanisms to focus on specific residues or regions that are important for the prediction task. Another approach involves the utilization of per-residue likelihood scores generated by the PLM. These scores, which reflect the probability of observing a particular amino acid at a given position, taking into account the context of the surrounding sequence, may indicate regions that are important for function or stability.

UniRep, an early example of a protein language model, utilizes LSTM and is trained on over 24 million protein sequences [52]. It can generate a 1900-dimensional embedding for any given protein sequence [53], providing valuable information for protein engineering tasks, such as predicting binding affinity, stability, and expression levels. ESM-1b is another powerful model that leverages the transformer architecture and is trained on over 250 million protein sequences [54]. It can generate 1280-dimensional embeddings and excels in tasks such as secondary structure prediction, contact map prediction, and remote homology detection [55]. ESM-2, a successor to ESM-1b, further improves its performance and generalizability [56]. When trained on a massive dataset of protein sequences, ESM-2 can predict the structure, function, and other properties from a sequence alone. This ability to capture fundamental aspects of protein biology makes it valuable for various antibody engineering applications.

Specialized protein language models have been developed specifically for antibodies. AntiBERTy, a BERT-based model, was trained on natural antibody sequences [57] and initially designed to understand antibody affinity maturation. AbLang, trained on a comprehensive dataset of antibody sequences in the Observed Antibody Space (OAS) database [58, 59], can restore missing residues in antibody sequences. Following these advancements, Kenlay et al. developed IgBert and IgT5, training on a massive dataset of over two billion unpaired antibody sequences and two million paired sequences from the OAS database [60]. The ability to handle both paired and unpaired antibody sequences makes these models superior to existing antibody and protein language models in terms of sequence recovery, affinity prediction, and expression prediction.

These protein- and antibody-specific language models have become invaluable tools in antibody engineering, enabling researchers to harness the power of deep learning to address complex problems in antibody design and optimization.

### Feature extraction from structures

While sequence-based characteristics are important, three-dimensional (3D) structural data can improve ML models for antibody engineering. Structural features provide valuable insights into the spatial arrangements and interactions that govern antibody function and biophysical properties.

Graphical protein structure representations are effective for featurization. In this framework, individual residues are treated as nodes, and their spatial relationships, such as distances, angles, and inter-residue contacts, are encoded as edges. This graph-based representation captures the intricate network of interactions within an antibody structure. Graph neural networks (GNNs), a class of deep learning models designed to operate on graph-structured data, can then be applied to ascertain rich representations from these antibody structure graphs. GNNs propagate and aggregate information along the edges, effectively capturing both local and global structural contexts relevant for predicting the epitopes of antibodies [61–63].

Recent advancements in protein language models have demonstrated their ability to integrate sequence and structure information. Unlike its predecessors, such as ESM-1b or ESM2, ESM3 explicitly incorporates 3D structural data during training [64]. This allows the model to learn a richer representation of proteins, capturing the intricate relationships between sequences, structures, and functions. ESM3 uses a discrete autoencoder to tokenize protein structures, representing them as a sequence of discrete tokens that capture the local structural neighborhoods around each amino acid. This innovative approach enables ESM3 to excel in both structure prediction and generation tasks, thereby demonstrating its potential for programmable protein engineering.

Integrating these structure-based featurization techniques with sequence-based approaches, including those employed in models such as ESM3, will lead to significant improvements in the prediction of various antibody properties. This will improve in silico screening and therapeutic candidate design. Recent advances have enabled the incorporation of 3D structural information into antibodies [65, 66]. Structure-aware pre-training enables the model develop meaningful representations that better capture these intricate antibody sequence‒structure relationships.

In addition, recent work has focused on developing methods for de novo design of proteins, particularly for binder design. RFdiffusion is a notable example, employing a diffusion-based generative model adaptable for antibody design [67]. This method allows for the generation of antibodies with desired structural features, such as specific CDR loop conformations or binding orientations, and has successfully generated single-domain antibodies and single-chain Fv (scFv) de novo [68]. AlphaProteo is another example that uses a diffusion-model-based approach to generate novel protein binders that target specific epitopes with high affinity [69]. While AlphaProteo was used to design de novo proteins that are not antibodies, its underlying diffusion-based approach could, in theory, be modified for antibody design, notably by focusing on CDR regions.

## Practical examples of data-driven antibody design

This section highlights the practical applications of data-driven methods in antibody engineering, demonstrating how ML is transforming antibody design and optimization (Table 4). These methods have been shown to increase binding affinity and to refine other properties that are critical for the efficacy and developability of antibodies. This enhances the connection between computational predictions and experimental validation.

**Table 4:** Recent publications reported on the design of antibodies utilizing machine learning approaches

Display or expression method	Screening method for binding	Parameter(s) for ML	Antibody feature(s) used for ML	Affinity validation	Outcome	References
Mammalian cell display	FACS	Antigen binding (binary)	One-hot encoding	BLI	30/30 variants predicted to bind HER2 retained binding specificity	[35]
Yeast display	FACS	Antigen binding, Nonspecific binding (binary)	One-hot encoding, PhysChem, UniRep	ELISA	EM2 variant showed increased antigen binding (1.28x; EC_50_ from 4.4 nM to 2.4 nM) and reduced non-specific binding (0.30x)	[34]
Yeast display	FACS	CDR3 sequence identity (clustering)	One-hot encoding	BLI	Multiple optimized VHH hits obtained from four clusters, showing high-affinity binding and favorable early developability profiles	[32]
Yeast display	FACS	Off-rate binning (Highest/Medium/Lowest)	Pre-trained autoencoder	BLI	This pipeline identified atezolizumab scFv mutants with better off-rates for PD-L1, with one mutant showing a tenfold decrease in the off-rate (from 6.3 × 10^–5^ s^−1^ to 6.5 × 10^–6^ s^−1^) and 17-fold improvement in affinity (*K*_D_ from 92 pM to 5.3 pM) with human PD-L1	[75]
Phage display	Phage panning	R2-to-R3 enrichment	One-hot encoding	ELISA	This model predicted enrichment of new sequences, designed higher affinity sequences, and improved antibody specificity by eliminating non-specific binders	[29]
Phage display	Phage panning	NLL (negative log-likelihood)	One-hot encoding	SPR	Affinity of generated sequences (*K*_D_ 42 nM) was over 1800-fold higher than that of the parental clone (*K*_D_ 77 µM)	[28]
Phage display	Phage panning	R2-to-R3 enrichment	One-hot encoding	BLI, ELISA	Computational counterselection outperformed molecular counterselection in removing off-target antibodies	[30]
*E. coli*	FACS, SPR	ACE score, *K*_D_, *k*_on_, *k*_off_	Pre-trained and finetuned RoBERTa model	SPR	Models designed sequences with desired binding properties (*K*_D_, *k*_on_, *k*_off_)	[70]
Yeast mating assay	Yeast mating	Predicted affinity calculated from mating efficiency	Pre-trained BERT model	Yeast mating	The best scFv generated from the ML approach (3.8 pM of predicted affinity) represents a 28.7-fold improvement in binding over the best scFv from directed evolution approach (109 pM of predicted affinity)	[74]
Public database	Public database	*ΔΔG*	Structure-guided metrics (amino acid interface score, significant interaction network score, Rosetta energy terms)	BLI, ELISA	Engineered antibodies showed up to > 1000-fold improved affinity (*K*_D_ 4.4 nM) compared to the corresponding template mAbs (*K*_D_ 2.0 pM) against various Omicron subvariants	[86]
-	-	Sequence likelihood	ESM-1b, ESM-1v (ensemble)	BLI	Improved the binding affinities of four clinically relevant, mature antibodies up to sevenfold (from 0.21 nM to 0.03 nM of *K*_D_) and three unmatured antibodies up to 160-fold (from 75 µM to 480 nM of *K*_D_)	[71]
-	-	Sequence likelihood	ESM-IF1	BLI	Achieved up to 25-fold improvement in neutralization (from 110 µg/µL to 4.3 µg/µL of IC_50_) and 37-fold improvement in affinity (from 46 µM to 1.2 µM of *K*_D_) against antibody-escaped viral variants of concern BQ.1.1 and XBB.1.5	[72]
-	-	Binding affinity, expression level	ESM-2	ELISA	Two antibodies with improved binding affinity and/or expression levels against diverse targets, including SARS-CoV-2 spike protein and human transferrin receptor	[76]

### Affinity maturation

A primary focus of data-driven antibody engineering is affinity maturation, which enhances the antibody binding strength. Traditionally, this process has been labor-intensive and relied on trial and error. However, AI-driven methods powered by large antibody datasets and advances in machine learning have enabled more efficient and rational approaches.

The integration of ML with high-throughput display technologies, such as phage and yeast display, has proven particularly powerful. These technologies allow for the rapid screening of vast antibody libraries, generating extensive datasets of antibody sequences and their corresponding binding affinities, which provide invaluable training data for ML models. For example, Mason et al. used deep neural networks to predict the antigen specificity of trastuzumab variants displayed on mammalian cells [35]. Their model, trained on data from FACS screening, successfully classified binders and non-binders. Specifically, they were able to identify 30 out of 30 predicted variants that retained binding to HER2, demonstrating a significant improvement in identifying HER2-specific subsets from a vast space of virtual variants. This exemplifies how deep learning can predict antibody specificity from sequence data, streamlining the screening of extensive libraries.

Similarly, Arras et al. combined yeast display, next-generation sequencing, and AI/ML to optimize humanized single-domain antibodies [32]. By analyzing sequence data, their approach rapidly identified potent VHH hits. Their work resulted in several optimized VHH hits from four different clusters that exhibited high-affinity binding and favorable early developability profiles. This study highlights the combined power of experimental and computational approaches to accelerate antibody optimization.

Other ML models have also demonstrated success in predicting and optimizing antibody affinity. Bachas et al. employed deep learning to predict binding affinities via high-throughput FACS- and SPR-based systems [70]. Their models accurately predicted the binding affinities of unseen variants across a large mutational space, demonstrating the potential of deep learning for the quantitative prediction of antibody‒antigen interactions. Their work also emphasized the importance of considering developability and immunogenicity during the design process by introducing "naturalness" as a metric for assessing variant similarity to natural immunoglobulins.

The recent development of protein language models (PLMs) trained on massive protein sequence databases has revolutionized antibody affinity maturation. These models capture the intricate relationships among sequence, structure, and function, enabling accurate and nuanced predictions for antibody design without the need for acquiring new, task-specific training data. For example, deep generative models based on PLM have been successfully applied to guide affinity maturation [71], leveraging the pre-trained knowledge embedded within the PLM to explore vast sequence spaces and identify high-affinity variants. This effectively reduces the dependence on costly and time-consuming experimental screenings. Furthermore, they provided another striking example of the power of structure-guided PLMs [72]. They utilized an inverse folding model, ESM-IF1 [73], augmented with structural information to guide the evolution of antibodies. This approach, when applied to two therapeutic antibodies against SARS-CoV-2, resulted in up to a 25-fold improvement in neutralization and a 37-fold improvement in the affinity for antibody-escaped viral variants. Crucially, this improvement was achieved by leveraging the structural information of the antibody**‒**antigen complex, showcasing the advantage over sequence-only based PLMs. This study highlights the value of incorporating structural information into PLMs for antibody optimization, thereby opening new possibilities for enhancing antibody function.

Combining language models with Bayesian optimization further enhances the effectiveness of affinity maturation. Li et al. integrated BERT language models with a yeast mating assay, achieving a 28.7-fold improvement in binding affinity compared with traditional methods [74]. Similarly, Parkinson et al. developed the RESP pipeline, which uses a pre-trained autoencoder and a variational Bayesian neural network to explore the sequence space and improve antibody affinity [75]. These hybrid approaches demonstrate the potential of combining ML techniques to achieve significant improvements in affinity maturation.

Beyond these approaches, recent work has demonstrated the potential of combining PLMs with active learning for rapid antibody optimization. Jiang et al. developed EVOLVEpro, a platform that integrates a PLM with a few-shot active learning strategy to improve antibody properties iteratively [76]. By focusing on a small number of experimental measurements in each round, EVOLVEpro was able to significantly enhance the binding affinity of antibodies against two targets.

### Beyond affinity: optimizing specificity, stability, and developability

Data-driven approaches are also instrumental in addressing antibody properties beyond affinity, which is crucial for therapeutic success. This includes optimizing specificity to minimize off-target binding and reduce potential side effects. Saksena et al. demonstrated a computational counterselection method using machine learning, surpassing traditional methods in identifying non-specific therapeutic biologic candidates [30]. Their approach, which trained on enrichment over rounds of panning in phage display experiments, showed that computational counterselection outperformed molecular counterselection in removing off-target antibodies.

Enhancing stability is also critical for developability and manufacturability. Harmarkar et al. successfully developed an ML model to predict the thermostability of scFv antibodies [47]. Using sequence and structural features, and validating their model with experimental measurements, they pinpointed key residue positions and mutations that enhanced stability. Similarly, Alvarez and Dean demonstrated the effectiveness of using protein embeddings, specifically those derived from the ESM-2 model, to predict the *T*_m_ of nanobodies [77]. Their tool, TEMPRO, achieved high accuracy in predicting the *T*_m_, offering a valuable resource for optimizing nanobody stability for various biomedical and therapeutic applications.

Addressing the challenge of high viscosity at high concentrations, which can hinder formulation and administration, is also possible with data-driven approaches. DeepSCM, a convolutional neural network model, can predict antibody viscosity solely on the basis of sequence information, offering a promising solution for streamlining formulation development [78]. Trained on a dataset of 6596 nonredundant antibody variable regions, DeepSCM achieved a linear correlation coefficient of 0.9 with experimental viscosity measurements, demonstrating its potential for high-throughput viscosity screening. Finally, ML holds immense potential for optimizing a wider spectrum of developability-related properties, such as aggregation propensity, solubility, and expression levels. Makowski et al. demonstrated this by constructing an interpretable ML model to identify antibody mutants with optimized non-specific binding and self-aggregation properties, providing a powerful tool to address critical developability challenges [79].

The continued development and application of data-driven approaches hold immense potential for designing and optimizing antibodies with improved binding affinity, specificity, stability, and overall developability.

## Conclusions

Driven by high-throughput experimental techniques and advanced ML methods, data-driven antibody engineering has remarkably progressed. This combination has accelerated the discovery and optimization of therapeutic antibodies, thereby addressing conventional empirical limitations.

The application of ML models to large-scale antibody datasets, including sequences, structures, and binding assay readouts, can accurately predict critical properties such as affinity, specificity, and developability. These capabilities enable researchers to rationally design antibodies and efficiently optimize existing leads.

High-throughput techniques, including NGS, display technologies, and biophysical assays, can be used to generate comprehensive datasets for the development of ML models. Advanced featurization strategies, such as protein language models and graph neural networks, effectively capture intricate sequence–structure–function relationships and enhance predictive performance. Emphasis on capturing both sequence and structural features is key to the success of these strategies.

The recent advancements in PLMs are particularly remarkable, enabling the proposition of effective sequence designs from limited data [71, 72, 76]. In this context, in-depth biophysical measurement techniques for individual clones, which have traditionally been used only for validation due to throughput limitations, are expected to become increasingly important as sources of training data.

Although considerable progress has been made, several issues still require attention. The intricacies of antibody–antigen interactions, particularly in the context of conformational epitopes and dynamics, necessitate the development of more sophisticated models that can accurately capture these nuances. Furthermore, the prediction of multiple properties such as immunogenicity and manufacturability requires further research.

In the future, the continued expansion of antibody datasets driven by collaborative efforts and data-sharing initiatives will be crucial for training more robust and generalized ML models. Moreover, the implementation of interpretable and explainable AI techniques is pivotal for elucidating the molecular determinants of antibody function and guiding rational engineering strategies.

Presently, many pharmaceutical companies are engaged in the acquisition of large-scale datasets, which they subsequently utilize to design therapeutic drugs with the aid of AI. For example, ABS-101, an anti-TL1A antibody designed via Absci's AI platform, has initiated an Investigational New Drug (IND) application for the treatment of inflammatory bowel disease and other diseases characterized by inflammation and fibrosis [80]. It is anticipated that this trend will persist. However, there are concerns that commercial companies with substantial investments in this field may exercise exclusive control over these datasets. In this context, there is a strong need for the further development of open, large-scale protein language models and methodologies that facilitate iterative, relatively small-scale experimentation to enhance target physical properties for the sustainable development of this research field.

In conclusion, data-driven antibody engineering has emerged as a transformative paradigm for revolutionizing the development of novel therapeutic antibodies. Capitalizing on the complementarity between high-throughput experimentation and ML, this approach offers a rational, efficient, and scalable framework to address the growing global demand for innovative biological drugs that can be used to treat diverse diseases. High-throughput experimentation plays a crucial role not only in generating the large-scale datasets required for training robust ML models but also in providing the necessary experimental validation of model predictions. The iterative cycle of computational design and experimental validation is key to the success of data-driven antibody engineering.

## Data Availability

Not applicable.

## Abbreviations

AI: Artificial intelligence
ML: Machine learning
NGS: Next-generation sequencing
CDRs: Complementarity determining regions
FACS: Fluorescence-activated cell sorting
BLI: Bio-layer interferometry
SPR: Surface plasmon resonance
DSC: Differential scanning calorimetry
DSF: Differential scanning fluorimetry
PSSMs: Position-specific scoring matrices
LSTM: Long short term memory
BERT: Bidirectional encoder representations from transformers
OAS: Observed antibody space
3D: Three-dimensional
GNNs: Graph neural networks
PLMs: Protein language models
